# Hospitals and the Duty of Business Firms

**Published:** 1903-07-18

**Authors:** 


### The Hospital.

A Journal of

The Medical Sciences and Hospital Administration.

Edited by Sir Henry Burdett, K.C.B.

Vol. XXXIV.—No. 877. [July 18, 1903.

### Hospitals and the Duty of Business Firms.

The *Times* of the 9th instant contains an excellent letter from Mr. J. G. Craggs, on subscriptions to London Hospitals. The duty of directors of limited companies and managers of mercantile houses in the matter is clear. The business aspects of this question are equally plain. Mr. Craggs points out in regard to the latter aspect that for a limited company or commercial house to open a charity account would be a personal act springing from high motives, but it is the duty of directors and managers to consider the matter from the pecuniary benefit to the undertaking alone. Let us then first consider the pecuniary benefit rendered at present by hospitals to the undertakings alone.

If the hospitals were not supported by voluntary contributions, they would have to be maintained out of the rates. Rate support would mean the addition of something like two million pounds per annum to the rates, which would have to be paid in large measure by the great companies and commercial houses within the metropolis. To show a little in detail what this would mean to each, Mr. Craggs quotes the case of the great railway companies. He refers to education formerly supported in large measure by voluntary subscriptions, afterwards rate aided, then fully charged on the rates, and at present State managed. He estimates that the hospital rate, if made, would before very long be about the same in amount as the present education rate. He shows that the great railway companies are now paying £100,000 per annum for education, that they have done so since 1870, and they will probably have to pay at least this sum in perpetuity. If the railway directors, and by inference all directors of companies, and managers of commercial houses in London, had voluntarily subscribed one-tenth of the sum they now have perforce to pay in rates for education, Mr. Craggs' contention is that a saving of £90,000 a year to railway shareholders and private firms might have been secured for many years, if not for all time. No doubt shareholders prior to 1870 might have censured the directors who so subscribed, but on the other hand the shareholders of to-day would readily have subscribed to a memorial to the former directors who made the original voluntary grants to education, when they realised that this step had saved their property from a perpetual charge of £90,000 per annum. The moral of this is, that private firms and public companies, and especially life insurance companies, who directly benefit from hospital work in innumerable ways, should take time by the forelock, and as a simple matter of business each vote a substantial annual subscription to the London hospitals. This subscription should be sent to King Edward's Hospital Fund at the Bank of England, because it affords an absolute guarantee to the subscribers, and at the same time saves directors of companies making a selection of individual hospitals, or dividing their payments into many parts.

Reverting next to the position of those public companies and private firms which receive direct benefit from the hospitals through the relief given to their employees, we know from experience, that each hospital has the control of this aspect of the question largely in its own hands. Good bookkeeping is the very essence of success in business. Our hospitals ought to so keep their books—and many of them do so—that it is easy for the management each year to make out an account showing how many patients, being members of the staff of a particular business, have been under treatment during the year, and the cost of such treatment, together with the annual subscription received by the hospital from the firm in question and its employees. A balance-sheet of this kind sent in to the board or managers of the particular undertaking each year has enabled several hospitals to raise their revenue to an adequate sum, and to immeasurably increase the efficiency of their administration.

The time is ripe for concerted action by the hospitals and King Edward's Hospital Fund, and we hope steps will be taken in the early autumn, and an active crusade instituted, so that the true bearing of this question and the relations between business firms and our voluntary hospitals may be fully recognised. Voluntary hospitals, and those who are interested in their management, should bring this question of insurance permanently before boards of directors and firms, and every opportunity should be taken to form the correct opinion upon it amongst their shareholders. The first step no doubt rests with the directors, whose duty it is to do all such things as may be for the benefit of their undertakings, and to provide against all risks. It is no doubt satisfactory that our hospitals have so far been supported by voluntary contributions. But the time has come when the points raised by "J. B." and Mr. Craggs should be made widely known, for then the question of rate support should be speedily settled in favour of the present system. Such a step may in fact save the property of shareholders and business firms from a perpetual charge for hospitals, similar to that which they now have to bear for education. Our rates are already too high, and the tendency is to constantly increase them; so that, as a mere matter of business, the sooner public companies and firms look this matter in the face, and deal with it practically, the better it will be for the interests entrusted to their care.

